# The Reminiscence Bump Effect in Autobiographies

**DOI:** 10.1093/geroni/igaa057.1182

**Published:** 2020-12-16

**Authors:** Hannah Benz, Thomas Pierce, Grace Flood

**Affiliations:** 1 Radford University, Blacksburg, Virginia, United States; 2 Radford University, Radford, Virginia, United States

## Abstract

The reminiscence bump effect refers to the tendency for older adults to recall more life events from their late teens through early thirties than from other periods of life. Participants in laboratory studies are often encouraged to recall events quickly and spontaneously, with the intention of eliciting those life events that come most easily to mind. The purpose of this study was to determine if a reminiscence bump effect is observed in accounts of life events that are carefully organized and narrated over prolonged periods of time; specifically, those presented in published autobiographies. 1911 life events were collected from the autobiographies of 16 authors. Four authors were female and 12 were male. The mean age of authors at the time of publication was 65.88 (SD = 13.94). 45.52% of life events reported in autobiographies occurred when authors were between 18 and 32 years of age, a value significantly higher than expected by chance [Chi-square (1, N = 1911) = 457.70, p < .001] and consistent with those found in laboratory studies supporting the reminiscence bump effect (e.g., Rubin, Wetzler, & Nebes, 1986). In addition, the 15-year reminiscence bump period represented an average of 22.77% of the authors’ lives at the time they published their autobiography; however, events from the reminiscence bump period took up an average of 42.52% of the pages in their autobiographies. These data provide evidence for a reminiscence bump effect in carefully considered life story narratives which required months or years to complete.

